# Use of the right internal mammary artery and the great saphenous vein for left anterior descending artery revascularization in patients whose left internal mammal artery cannot be used: a study based on transit-time flow measurement

**DOI:** 10.1186/s13019-020-01172-5

**Published:** 2020-06-05

**Authors:** Guodong Zhang, Zhou Zhao, Yu Chen, Shenglong Chen, Gang Liu

**Affiliations:** Cardiac Surgery Department, Peking University People’s Hospital, BeiJing, 100044 China

**Keywords:** Left descending artery, Transit-time flow measurement, Internal mammal artery, Great saphenous vein

## Abstract

**Background:**

Owing to the high patency, the use of the left internal mammary artery (LIMA) for left anterior descending artery (LAD) grafting has been a cornerstone of coronary artery bypass grafting surgery (CABG). However, for some patients whose LIMA cannot be used, surgeons have to choose other conduit materials to revascularize the LAD. The purpose of this study was to explore the differences in different conduit materials used for LAD in terms of parameters measured by transit-time flow measurement (TTFM) and the early graft patency detected by computed tomography angiography.

**Methods:**

We retrospectively collected the data of 410 patients who underwent isolated primary OPCAB with intraoperative TTFM data. According to the strategy of the LAD revascularization, 410 patients were assigned to three groups: a left internal mammal artery (LIMA) group (*n* = 333), a right IMA (RIMA) group (*n* = 34) and a great saphenous vein (SVG) group (*n* = 43). The baseline and perioperative blood parameters were compared for the three groups, as well as the early graft patency rates.

**Results:**

Compared with the LIMA-LAD group, the SVG-LAD group had a significantly higher mean graft flow volume (MGF) (37.15 ± 23.29 vs 29.71 ± 20.94 ml/min, *P* = 0.036), however, had a lower value of pulse index (PI) (2.07 ± 0.62 vs 2.65 ± 1.01, *P*<0.001). There was no significant difference between the two groups in terms of DF (*P*>0.05). Compared with the RIMA-LAD group, the SVG-LAD group just had a lower value of PI (2.07 ± 0.62 vs 2.56 ± 0.96, *P* = 0.029). However, there was no significant difference between the two groups in terms of MGF and DF (*P*>0.05). Compared with the LIMA-LAD group, the RIMA-LAD group had a slightly lower value of DF (70.76 ± 11.87 vs 74.06 ± 7.09, *P* = 0.018), while there was no difference in terms of MGF and PI between the two groups (*P*>0.05). The patency rate of the LIMA-LAD group was 98.72% (309/313), that of RIMA-LAD group was 97.06% (33/34), and that of SVG-LAD group was 100%. There was no difference among the three groups in patency rate (*P* = 0.405).

**Conclusions:**

SVG-LAD has a higher intraoperative MGF and a lower value of the PI than both LIMA-LAD and RIMA-LAD. RIMA has a higher preoperative blood flow and a lower value of the PI than LIMA; however, there were no significant difference between RIMA grafted to LAD and LIMA grafted to LAD in terms of MGF, PI and DF. In situ skeletonized RIMA did not increase blood flow compared to pedicled LIMA.

## Background

Coronary artery disease remains the most common cause of death worldwide, responsible for about one in every seven deaths [1]. Since Robert Goetz first performed and published the coronary artery bypass graft surgery in humans in 1961, now CABG has become an important revascularization methods of coronary heart disease [2]. Since the mid-1980s, owing to the high patency, the use of the left internal mammary artery (LIMA) for left anterior descending artery (LAD) grafting has been a cornerstone of CABG surgery [3]. However, owing to stenosis or occlusion of LIMA or harvested damage, surgeons have to choose other conduit materials to revascularize the LAD. Transit-time flow measurement (TTFM) has been increasingly applied to judge the quality of anastomosis and blood flow during CABG, because TTFM is less invasive, more reproducible, and less time consuming [4]. A previous study demonstrated that studies on TTFM should consider conduit materials and different coronary territories, while few studies on the comparison of different conduit materials used for LAD in terms of TTFM parameters have been reported [5]. The purpose of this study was to explore the differences in different conduit materials used for LAD in terms of parameters such as the pulse index (PI), mean graft flow (MGF) and diastolic flow fraction (DF) measured by TTFM.

## Material and methods

### Study population

**Fig. 1:**
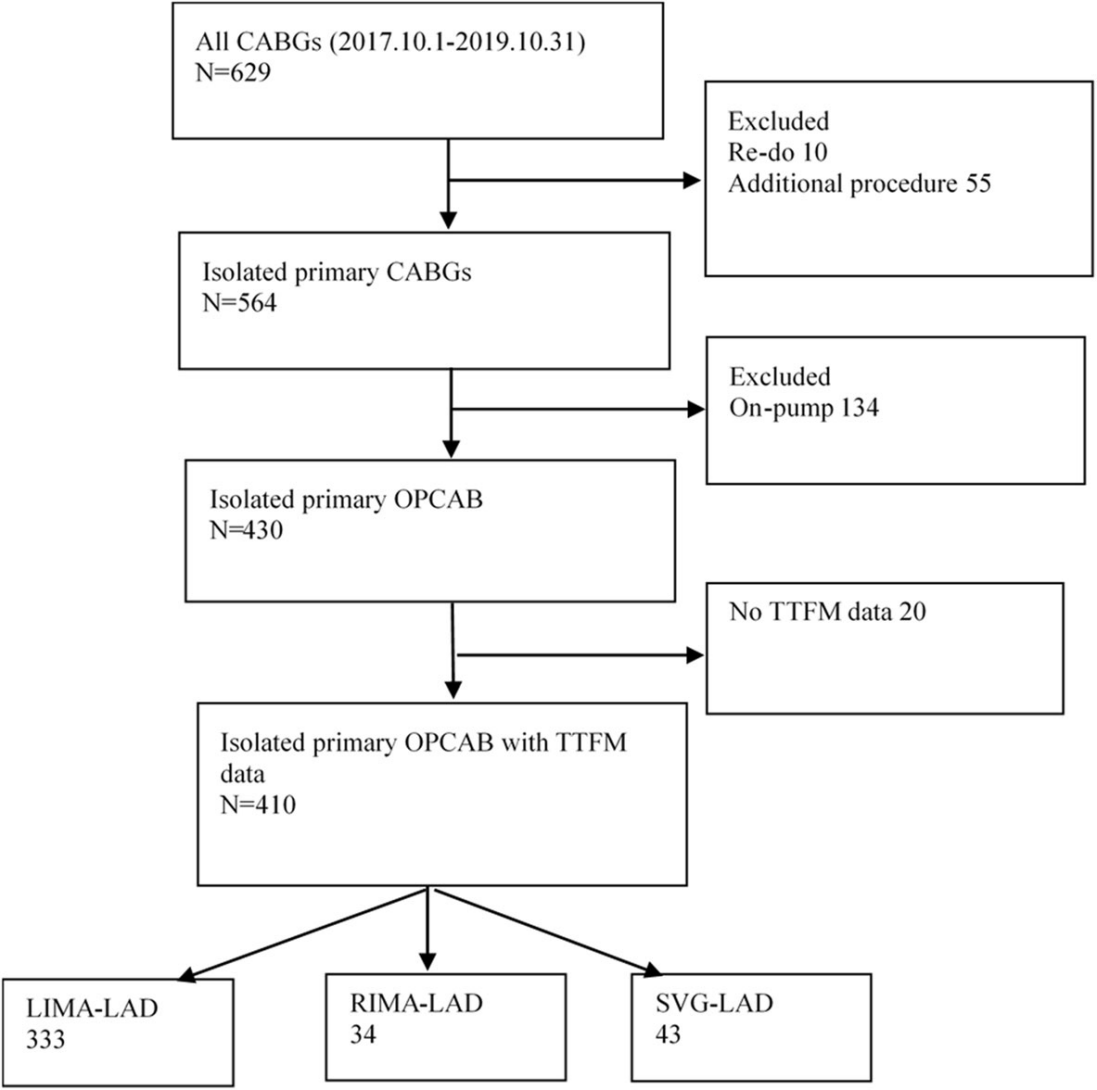
Data flowchart for patients included in the study. CABG, Coronary artery bypass grafting; LAD, left anterior descending artery; LIMA, left internal mammary artery; RIMA, right internal mammary artery; TTFM, transit-time flow measurement

Data for isolated CABG were retrospectively collected from October 1, 2017 to October 31, 2019, from the Peking University People’s Hospital database. There were 629 patients who underwent CABGs; we excluded 10 who underwent redo surgeries, 55 who underwent concomitant additional procedures, 134 who underwent on-pump CABGs and 20 without intro-operative TTFM data (Fig. 1). We included 410 patients who undergoing isolated primary OPCAB with intro-operative TTFM data. According to the strategy of the LAD revascularization, 410 patients were assigned to three groups: a LIMA group (*n* = 333), a RIMA group (*n* = 34) and a great saphenous vein (SVG) group (*n* = 43). This study was approved by our institutional Review Board /Ethics Committee. Consent for individual use of data was waived because of the nature of the study and previous approval for the use of such data at the time of operative consent.

### Surgical methods

All patients underwent OPCAB through a median full sternotomy. Heparin was given to reach an activated clotting time > 300 s. The central temperature was maintained above 36 °C. The pericardium was opened and suspended to expose the heart and the heart was displaced using a posterior pericardial stitch and gauze swabs. Patients lacking good presentation of the target arteries on the lateral and inferior aspect of the heart were placed in a gentle right decubitus Trendelenburg position to assist in visualization. Stabilization of the target coronary arteries was accomplished with a tissue stabilizer (Octopus, Medtronic Corporation, Minneapolis, MN). An intra-coronary shunt (Medtronic Corporation, Minneapolis, MN) was used during grafting in most operations. In this study, the RIMAs were all harvested by the skeletonized method, and the LIMAs were all harvested by the pedicle method. The graft anastomosis was secured using a 7–0 or 8–0 polypropylene suture.

### Preoperative internal mammary artery ultrasonography examination

Preoperative blood flow parameters of IMAs were measured by transthoracic doppler ultrasonography machine (APLIO500 TUS-A500, Probe: PLT-704SBT and PVT-712BT). All preoperative internal mammal artery ultrasonography was performed by the same senior ultrasonologist.

### Intraoperative transit time flow measurement

The transit time flow of the grafts was measured by The VeriQ system TTFM device (MediStim Inc., Oslo, Norway), equipped with 2, 3 or 4-mm probes, depending on the size of the graft, under stable haemodynamic conditions without the support of a mechanical device such as cardiopulmonary bypass or an intra-aortic balloon pump. The parameters yielded by TTFM system including (i) the mean graft flow volume (MGF, ml/min), (ii) the pulse index calculated as (PI, maximum flow volume—minimum flow volume)/(mean flow volume) and (iii) the DF, calculated as (flow volume of the diastolic phase)/(flow volume of the systolic phase + flow volume of the diastolic phase). Satisfactory blood flow parameters criteria: 1, ACI>50%; 2, The shape of blood flow waveform is stable and repeatable; 3, PI<5, MGF>15 ml/min. If sufficient graft flow was not obtained, graft revision was considered and performed until diastolic graft flow was confirmed.

### Postoperative management

Postoperatively, aspirin, nitroglycerine and β- blocker were prescribed on postoperative day 1. The patients were routinely examined by cardiac CT scanning prior to discharge from the hospital unless they had grade 3 or more chronic kidney disease.

### Statistical analysis

The database was established by EpiDate3.1 software, the data were input twice in parallel. The final analysis database is formed after logical error checking and sorting of the input data and analysis and processing of outliers. Continuous variables were expressed as means ±SDs; if the data conformed to a normal distribution, the two groups were compared using an independent samples t test, and multiple groups were compared using variance analysis. The least significant difference (LSD) was used for pairwise comparisons among those with intragroup differences. For nonnormally distributed data, Wilcoxon rank-sum tests were used for comparisons between two groups, and Kruskal-Wallis H tests were used for comparison between multiple groups. Categorical variables were described as percentages (rates); comparisons between two groups were performed using chi-square tests, and comparisons between multiple groups were performed using crosstabulation analysis. *P* < 0.05 was considered statistically significant. All analyses were performed in SPSS version 23.

## Results

As shown in Table 1, patients in the RIMA group were younger and were less likely to have a history with diabetes than the other two group. There was no difference in the other data. The reasons the LIMA cannot be used are shown in Table 2.

**Table 1:** Demographic and clinical characteristics

	LIMA-LAD (333)	RIMA-LAD (34)	SVG-LAD (43)	*P*
Male (n, %)	241(72.37)	29(85.29)	30(69.77)	0.110
Age	64.27 ± 10.70	56.85 ± 11.01	70.47 ± 10.25	0.000
BMI	26.64 ± 20.25	25.55 ± 2.51	23.79 ± 3.09	0.616
Hypertension (n, %)	209(62.76)	21(61.76)	27(62.79)	0.152
Diabetes (n, %)	141(42.34)	13(38.24)	17(39.53)	0.036
Hyperlipidemia (n, %)	145(43.54)	15(44.12)	19(44.19)	0.455
Previous stroke (n, %)	50(15.01)	9(26.47)	6(13.95)	0.158
COPD (n, %)	7(2.10)	1(2.94)	2(4.65)	0.451
PVD (n, %)	40(12.01)	6(17.65)	3(8.82)	0.723
Previous infarction (n, %)	65(19.52)	6(17.65)	7(16.28)	0.730
PCI	49(14.71)	6(17.65)	8(18.60)	0.719
Number of anastomosis	3.04 ± 0.94	2.78 ± 0.87	3.24 ± 1.07	0.121
NYHA	2.30 ± 0.51	2.33 ± 0.60	2.28 ± 0.51	0.911
LVEF (%, ±s)	62.87 ± 25.18	62.81 ± 9.50	61.40 ± 8.78	0.927
LVEDd (cm, ±s)	50.69 ± 7.034	50.25 ± 6.201	51.43 ± 6.64	0.741

*BMI* Body mass index, *COPD* Chronic obstructive pulmonary disease, *PVD* peripheral vascular diseases, *PCI* Percutaneous coronary intervention, *NYHA* New York Heart Association, *LVEF* left ventricular ejection fraction, *LVEDd* Left ventricular end-diastolic dimension, *LIMA* Left internal mammary artery, *SVG* Great saphenous vein, *RIMA* Right internal mammary artery, *LAD* Left anterior descending artery

**Table 2:** The reason about the LIMA cannot be used

Reason	Number
Poor flow after harvesting	20
The proximal stenosis	24
Diameter < 1.5mm	5
Harvesting damage	15
Stolen blood syndrome	6
Other reason	7

### The comparison of preoperative blood flow parameters between LIMA and RIMA

Compared with the LIMA, the RIMA had a slightly larger diameter (1.97 ± 0.19 vs 1.96 ± 0.17 mm, *P* = 0.008) and a larger mean graft flow volume (18.68 ± 8.75 vs 16.48 ± 7.13 ml/min, *P*<0.001), but a lower value of pulse index (3.97 ± 1.20 vs 4.13 ± 1.36, *P* = 0.002) (Table 3).

**Table 3:** The blood flow parameters of LIMA and RIMA

Items	Group A	Group B	P
	Preoperative LIMA	Preoperative RIMA	
PI	4.13 ± 1.36	3.97 ± 1.20	0.002
MGF (ml/min)	16.48 ± 7.13	18.68 ± 8.75	0.000
Diameters (mm)	1.96 ± 0.17	1.97 ± 0.19	0.008
	Preoperative LIMA	Intraoperative LIMA	
PI	4.134 ± 1.36	2.65 ± 1.01	0.000
MGF (ml/min)	16.48 ± 7.13	29.71 ± 20.94	0.000
	Preoperative RIMA	Intraoperative RIMA	
PI	3.97 ± 1.20	2.56 ± 0.96	0.000
MGF (ml/min)	18.68 ± 8.75	29.03 ± 22.73	0.011

*LIMA* Left internal mammary artery, *SVG* Great saphenous vein, *RIMA* Right internal mammary artery, *MGF* Mean graft flow, *PI* Pulse index, *DF* Diastolic flow fraction

Compared with preoperative mean graft flow volume examined by ultrasonography, the intraoperative mean graft flow volume measured by TTFM of LIMA (29.71 ± 20.94 vs 16.48 ± 7.13 ml/min, *P*<0.001) and RIMA (29.03 ± 22.73 vs 18.68 ± 8.75, *P* = 0.011) are both higher, but the values of PI are both lower (*P*<0.001) (Table 3).

### The comparison of blood flow parameters examined by TTFM of different conduit grafting methods used for LAD revascularization

Compared with the IMAs-LAD group (LIMA-LAD+RIMA-LAD), the SVG-LAD group had a significantly higher mean graft flow volume (37.15 ± 23.29 vs 29.65 ± 21.08 ml/min, *P* = 0.033), but a lower value of pulse index (2.07 ± 0.62 vs 2.64 ± 1.00, *P*<0.001). There was no significant difference between the two groups in terms of DF (*P*>0.05) (Table 4).

**Table 4:** Intraoperative blood flow parameters in the three groups

Items	Group A	Group B	P
	IMAs-LAD (367)	SVG-LAD (43)	
PI	2.64 ± 1.00	2.07 ± 0.62	0.000
MGF (ml/min)	29.65 ± 21.08	37.15 ± 23.29	0.033
DF (%)	73.75 ± 7.71	71.85 ± 8.38	0.146
	LIMA-LAD (333)	RIMA-LAD (34)	
PI	2.65 ± 1.01	2.56 ± 0.96	0.642
MGF (ml/min)	29.71 ± 20.94	29.03 ± 22.73	0.859
DF (%)	74.06 ± 7.09	70.76 ± 11.87	0.018
	LIMA-LAD (333)	SVG-LAD (43)	
PI	2.65 ± 1.01	2.07 ± 0.62	0.000
MGF (ml/min)	29.71 ± 20.94	37.15 ± 23.29	0.036
DF (%)	74.06 ± 7.09	71.85 ± 8.38	0.091
	RIMA-LAD (34)	SVG-LAD (43)	
PI	2.56 ± 0.96	2.07 ± 0.62	0.029
MGF (ml/min)	29.03 ± 22.73	37.15 ± 23.29	0.102
DF (%)	70.76 ± 11.87	71.85 ± 8.38	0.551

*LIMA* Left internal mammary artery, *SVG* Great saphenous vein, *RIMA* Right internal mammary artery, *MGF* Mean graft flow, *PI* Pulse index, *DF* Diastolic flow fraction

Compared with the LIMA-LAD group separately, the SVG-LAD group also had a significantly higher mean graft flow volume (37.15 ± 23.29 vs 29.71 ± 20.94 ml/min, *P* = 0.036), but a lower value of pulse index (2.07 ± 0.62 vs 2.65 ± 1.01, *P*<0.001). There was no significant difference between the two groups in terms of DF (*P*>0.05) (Table 4).

Compared with the RIMA-LAD group separately, the SVG-LAD group had only a lower value of pulse index (2.07 ± 0.62 vs 2.56 ± 0.96, *P* = 0.029). However, there was no significant difference between the two groups in terms of MGF and DF (*P*>0.05) (Table 4).

Compared with the LIMA-LAD group, the RIMA-LAD group had a slightly lower DF (70.76 ± 11.87 vs 74.06 ± 7.09, *P* = 0.018), while there was no difference in terms of MGF and PI between the two groups (*P*>0.05) (Table 4).

### Results of the CT angiography examined before discharge in the three groups

A total of 406 patients (313 patients in the LIMA-LAD group, 34 patients in the RIMA-LAD group and 43 patients in the SVG-LAD group) were examined for coronary CT angiography before discharge. Table 5 lists the coronary CT angiographic results. The patency rate of the LIMA-LAD group was 98.72% (309/313), that of the RIMA-LAD group was 97.06% (33/34), and that of the SVG-LAD group was 100%. There was no difference among the three groups in the patency rate (*P* = 0.405).

**Table 5:** Graft Patency in the three groups

Items	LIMA-LAD	RIMA-LAD	SVG-LAD	P
CTA	94.00(313/333)	100(34/34)	93.02(40/43)	0.381
Patency rate (%)	98.72(309/313)	97.06(33/34)	100(40/40)	0.405

*LIMA* Left internal mammary artery, *SVG* Great saphenous vein, *RIMA* Right internal mammary artery, *CTA* Computed tomography angiography

## Discussion

The strategy of in situ LIMA grafting to the LAD (LIMA-LAD) is considered the “gold standard” of coronary revascularization [6]. However, in some circumstances, such as the stenosis or occlusion of the LIMA and harvested damage, surgeons must choose other conduit materials to revascularize the LAD. There is no consensus on which conduit is better for LAD under an unusable LIMA circumstance. In situ or free RIMA and SVG are used considering the patients’ age, state of cardiac function and the surgeons’ preference. Previous study results demonstrated that revascularization of the LAD using an in situ RIMA resulted in excellent mid-term graft patency and clinical outcomes [7]. SVG are still widely used because of their several advantages, including ease of access, ease of operation, sufficiency of length for transplantation, and short harvest time. There is still no consensus on whether SVG grafted to the LAD could improve the flow compared with IMA grafts. Some studies have suggested that SVG grafted to the LAD has a higher blood flow (up to 35%) than IMA grafts, but other studies have suggested that there is no significant difference between SVGs and IMAs in terms blood flow [8, 9]. This study similarly showed that usage of SVG to bypass to the LAD has the advantage of higher MGF and a lower value of the PI compared with RIMA and LIMA during operation. The possible explanation may be that SVGs have larger diameters and are often not be affected by vasoactive drugs and neurohumoral fluids compared to arterial conduits. However, the explanation that SVG anastomosed directly to the ascending aorta with higher pressure and a higher low-gradient can cause a larger mean blood flow volume still lacks of assertive evidence. Previous studies have shown that the flow of the IMA graft in Chinese people is lower than that in western people which may be related to the fact that the diameter of IMA graft in Chinese people is smaller [10]. Our previous study also found that the intraoperative blood flow of an in situ IMA graft was close to the blood flow in other related research results, but the flow of the LIMA would increase significantly 1 week postoperatively, which was considered to be related to the intraoperative use of vasoactive drugs and self-regulation [11].

Previous studies have demonstrated that studies on TTFM should consider arterial versus venous grafts and different coronary territories [5]. However, owing to the high patency, the use of the LIMA for LAD grafting has been a cornerstone of CABG surgery; thus, few studies have compared the TTFM parameters of different conduits used for LAD. Therefore, we aimed to compare the TTFM parameters of different conduits used for LAD revascularization.

This study analyzed the preoperative ultrasound data of LIMA and RIMA, and found that the RIMA had a slightly larger diameter and a larger mean graft flow volume, but a smaller pulse index than the LIMA. One possible explanation may be that Chinese people are mainly right-handed and the muscles and blood vessels on the same side are developed better than those on the left side. However, the advantage of blood flow in RIMA was distinguished after IMAs bypassed to the LAD, and there was no difference of the MGF and PI assessed by TTFM between the LIMA-LAD group and the RIMA-LAD group during operation.

In the 1980s, Keeley SB first proposed the method of skeletonized harvesting of IMA, which can help to decrease the deep sternal infection caused by the simultaneous harvest of bilateral IMAs and can improve the utilization rate of bilateral IMAs [12, 13]. Some studies have shown that skeletonized-harvested IMAs extended the usable length, and removal of the peripheral tissues around the grafts could be beneficial for graft dilatation, which can increase the blood flow [14]. In this study, the RIMAs were all harvested by skeletonized method, and the LIMAs were all harvested by pedicle method, but there was no statistical significance in blood flow between them.

This study compared the blood flow parameters of IMAs between those examined preoperatively and those assessed intraoperatively, and the results demonstrated that compared with the preoperative data, the MGF values of both LIMA and RIMA were improved, the PI values of both LIMA and RIMA were decreased (*P*<0.001). The internal mammary artery arises from the undersurface of the first portion of the subclavian artery, and its distal end is the capillary network of the chest wall. Blood flow in the un-grafted LIMA occurs mainly during systole similar to the flow in peripheral arteries, like in the subclavian artery. Once grafted to the coronary network, the IMA flow pattern instantly adapts to the left ventricular hemodynamic. The diastolic flow velocity of the LIMA increases after CABG as a result of the physiologically decreased resistance in the coronary circulation. This “diastolization” of the IMA blood flow is also related to the low resistance and large capacitance of the coronary artery network and to the IMA self-regulated property on vascular tone [15, 16].

The PI, calculated as (maximum flow volume-minimum flow volume)/(mean flow volume), is one of the TTFM measurements parameters that used for conduit evaluation during operation [17]. The results of Di Giammarco et al. study showed that the PI>5 may be an independent risk factors of graft dysfunction. Higher PI values indicate that there may be greater negative flow or lower average flow [18]. The results of this study demonstrated that the PIs of both the LIMA-LAD group and the RIMA-LAD group are higher than those of the SVG-LAD group (*P*<0.001). We also found that in this study, the proportion of negative flow less than 10 ml/min was larger in the arterial conduit group (*P* < 0.001), suggesting that there was more negative blood flow, i.e., competitive flow, in the early systolic in the arterial conduit group. In contrast, the venous conduits have less smooth muscle, low elasticity and small cyclical deformation of pipe diameter with pressure. Therefore, it is impossible to accommodate the reverse flow by adjusting the diameter of the conduits, and the probability of the occurrence of competitive flow may be relatively low [3].

Owing to some patients do not have symptoms or clinical signs of myocardial ischemia prior to discharge, few studies about the acute asymptomatic graft failure have been reported and graft failure rates remain unclear [19]. However, early asymptomatic graft failure may have negative impact on the patients’ short- and long-term outcomes and develop symptoms when exercise increase, because the relevant myocardial area are still unsupplied [20–22]. Cardiac CTA, as a low-invasive investigation method for the evaluation of the early grafts has been proved to be another choice besides coronary artery angiography in several studies [22, 23].CTA examination, as a part of the graft quality evaluation study in our center, was routinely used in patients who underwent CABG prior discharge. In our study, the early patency before discharge of RIMA-LAD and SVG-LAD are comparable with that of the LIMA-LAD; however considering the long-term patency, the LIMA and RIMA were recommended over SVG.

### Limitations

Several limitations of our study should be recognized. The first and most important limitation of this study was its descriptive nature, using a relatively small cohort of patients at a single institution. Second, blood flow parameters measured by TTFM and early prior to discharge graft patency do not reflect the all of the advantages of the effect of the grafting strategy; in addition, major cardiovascular and cerebrovascular adverse events, revascularization events and the long-term graft patency can reflect the advantage of grafting strategy to the LAD. These other indicators were not included in this study.

## Conclusions

SVG-LAD has a higher intraoperative MGF and a lower value of the PI than both LIMA-LAD and RIMA-LAD. RIMA has a higher preoperative blood flow and a lower value of the PI than LIMA; however, there were no significant difference between RIMA grafted to LAD and LIMA grafted to LAD in terms of MGF, PI and DF. In situ skeletonized RIMA did not increase blood flow compared to pedicled LIMA.

## Data Availability

Data will be made available on request.

## Abbreviations

LIMA: Left internal mammary artery
LAD: Left anterior descending artery
CABG: Coronary artery bypass grafting surgery
TTFM: Transit-time flow measurement
PI: Pulse index
MGF: Mean graft flow volume
DF: Diastolic flow fraction
SVG: Great saphenous vein
CTA: Computed tomography angiography

## References

[1] Voudris KV, Kavinsky CJ (2019). Advances in Management of Stable Coronary Artery Disease: the role of revascularization?. Curr Treat Options Cardiovasc Med.

[2] EAV R (2017). Fifty years of coronary artery bypass graft surgery. Braz J Cardiovasc Surg.

[3] Gaudino M, Antoniades C, Benedetto U, Deb S, Di Franco A, Di Giammarco G (2017). Mechanisms, consequences, and prevention of coronary graft failure. Circulation.

[4] Takami Y, Takagi Y (2018). Roles of transit-time flow measurement for coronary artery bypass surgery. Thorac Cardiovasc Surg.

[5] Amin S, Madsen PL, Werner RS, Krasopoulos G, Taggart DP (2019). Intraoperative flow profiles of arterial and venous bypass grafts to the left coronary territory. Eur J Cardiothorac Surg.

[6] Benedetto U, Amrani M, Gaer J, Bahrami T, de Robertis F (2014). The influence of bilateral internal mammary arteries on short- and long-term outcomes: a propensity score matching in accordance with current recommendations. J Thorac Cardiovasc Surg.

[7] Ji Q, Xia L, Shi Y, Ma R, Shen J, Lai H (2017). Mid-term graft patency of right versus left internal mammary artery as arterial conduit usage for left anterior descending artery revascularisation: insights from a single-Centre study of propensity-matched data. Int J Surg.

[8] Hassanein W, Albert AA, Arnrich B, Walter J, Ennker IC, Rosendahl U (2005). Intraoperative transit time flow measurement: off-pump versus on-pump coronary artery bypass. Ann Thorac Surg.

[9] Leong DKH, Ashok V, Nishkantha A, Yue HS, Sim EKW (2005). Transit-time flow measurement is essential in coronary artery bypass grafting. Ann Thorac Surg.

[10] 高长青, 张涛, 李伯君, 肖苍松, 吴扬, 马晓辉: 中国人移植的乳内动脉平均流量测定及影响因素. *中国胸心血管外科临床杂志* 2004;011(2):84–7.

[11] 赵舟, 高俊雪, 秦俊超, 刘晶, 刘刚, 陈生龙, 陈彧: 经胸多普勒超声心动图观察冠状动脉旁路移植术围术期左乳内动脉-左前降支桥血管的血流变化. *中国循环杂志* 2018(7):690–3.

[12] Keeley S (1987). B: the skeletonized internal mammary artery. Ann Thorac Surg.

[13] Takami Y, Ina H (2002). Effects of skeletonization on intraoperative flow and anastomosis diameter of internal thoracic arteries in coronary artery bypass grafting. Ann Thorac Surg.

[14] Oliveira SMPB, Ferraz CPE, SHJdA C, Freire SA, MRG A, Lopes AM (2015). Flow capacity of skeletonized versus pedicled internal thoracic artery in coronary artery bypass graft surgery: systematic review, meta-analysis and meta-regression. Eur J Cardiothorac Surg.

[15] Hata M, Raman JS, Shiono M, Sezai A, Negishi N, Sezai Y (2002). What can Doppler wave forms of the left internal thoracic artery teach us? -the efficacy of apical transthoracic approach of Doppler echocardiography. Ann Thorac Cardiovasc Surg.

[16] Hirata N, Asaoka N, Amemiya A, Hatsuoka S, Ueno T, Kosakai Y (2003). Noninvasive evaluation of internal thoracic artery and left anterior descending coronary artery anastomotic sites using transthoracic Doppler echocardiography: comparison with coronary arteriography. J Thorac Cardiovasc Surg.

[17] Kieser TM, Rose S, Kowalewski R, Belenkie I (2010). Transit-time flow predicts outcomes in coronary artery bypass graft patients: a series of 1000 consecutive arterial grafts☆. Eur J Cardiothorac Surg.

[18] Di Giammarco G, Pano M, Cirmeni S, Pelini P, Vitolla G, Di Mauro M (2006). Predictive value of intraoperative transit-time flow measurement for short-term graft patency in coronary surgery. J Thorac Cardiovasc Surg.

[19] Zientara A, Rings L, Bruijnen H, Dzemali O, Odavic D, Haussler A (2019). Early silent graft failure in off-pump coronary artery bypass grafting: a computed tomography analysisdagger. Eur J Cardiothorac Surg.

[20] Hess CN, Lopes RD, Gibson CM, Hager R, Wojdyla DM, Englum BR (2014). Saphenous vein graft failure after coronary artery bypass surgery: insights from PREVENT IV. Circulation.

[21] Bassri H, Salari F, Noohi F, Motevali M, Abdi S, Givtaj N (2009). Evaluation of early coronary graft patency after coronary artery bypass graft surgery using multislice computed tomography angiography. BMC Cardiovasc Disord.

[22] Yoo K (2003). The comparison of the graft patency after coronary artery bypass grafting using coronary angiography and multi-slice computed tomography. Eur J Cardiothorac Surg.

[23] Anders K, Baum U, Schmid M, Ropers D, Schmid A, Pohle K (2006). Coronary artery bypass graft (CABG) patency: assessment with high-resolution submillimeter 16-slice multidetector-row computed tomography (MDCT) versus coronary angiography. Eur J Radiol.

